# Mixed-methods, cross-sectional assessment of a client-facing, family planning counseling chatbot in Côte d’Ivoire

**DOI:** 10.1093/oodh/oqae027

**Published:** 2024-08-03

**Authors:** Kristen M Little, Ndola Prata, Kpebo Djoukou Olga Denise, Nadia Tefouet, Jean Christophe Fotso, Alexandra Angel, Sarah Brittingham, Paul Bouanchaud, Amadou Donapoho Soro, Lisa Dulli

**Affiliations:** Strategy and Insights Department, Population Services International (PSI), Washington, DC, USA; Reproductive, Maternal, Newborn, Child and Adolescent Health (RMNCAH), Evidence for Sustainable Human Development (EVIHDAF), Yaounde, Cameroon; School of Public Health, University of California Berkeley, Berkeley, CA, USA; Unit of Research in Maternal and Child Health, National Institute of Public Health, Abidjan, Côte d'Ivoire; Reproductive, Maternal, Newborn, Child and Adolescent Health (RMNCAH), Evidence for Sustainable Human Development (EVIHDAF), Yaounde, Cameroon; Reproductive, Maternal, Newborn, Child and Adolescent Health (RMNCAH), Evidence for Sustainable Human Development (EVIHDAF), Yaounde, Cameroon; Sexual and Reproductive Health Department, PSI, Washington, DC, USA; Scientific, Technical and Evidence Advancement Department, FHI 360, Durham, NC, USA; Strategy and Insights Department, Population Services International (PSI), Washington, DC, USA; Strategic Information Department, PSI/Côte d’Ivoire, Abidjan, Côte d’Ivoire; Global Health and Population Research Department, FHI 360, Durham, NC, USA

**Keywords:** digital, chatbot, family planning, counseling, West Africa

## Abstract

Evidence is limited about the feasibility, acceptability and effects on family planning (FP) intentions of digital tools, such as chatbots. To help fill this gap, we assessed a chatbot in Côte d’Ivoire with three components: (i) menstrual health information; (ii) FP information and (iii) method recommendations and referrals. We conducted a cross-sectional study to describe user characteristics and perceptions of chatbot usability, content and relevance, and perceived effects on FP-related self-efficacy. We used a mixed-methods approach, including an online survey and in-depth interviews (IDI) with chatbot users, and chatbot back-end data. Qualitative data were analyzed thematically. Quantitative data were analyzed descriptively. Between 11 January 2022 and 15 July 2022, we recruited 280 chatbot users for the survey, of whom 28 completed an in-depth interview. Survey participants averaged 22 years old, were majority female (96%), resided in Abidjan (71%) and had some university education (61%). Less than half (48%) were currently using a method to delay/avoid pregnancy. Participants primarily accessed the chatbot to get information for themselves (73%) and felt it was ‘easy’ (29%) or ‘very easy’ to use (65%). Most respondents somewhat or strongly agreed that the information provided was accurate/reliable (97%) and was useful to them (96%). Respondents reported feeling more confident in discussing FP topics with their partners and providers after interacting with the chatbot. Our findings suggest that chatbots may be a feasible and acceptable approach to sharing tailored FP information with end-users in a West African context, especially for young women in urban areas.

## INTRODUCTION

In the context of high unmet need for family planning (FP) and low access to FP-related information, a variety of strategies are needed to get high-quality, accurate and user-friendly information to women and couples through a range of communication channels. Digital health for social and behavioral change interventions has been identified as a promising High Impact Practice for FP, to support, maintain and adopt healthy sexual and reproductive health (SRH) behaviors [1]. Interventions that increase understanding of contraceptive choices, including through quality counseling that reviews method and side effect information that is important to users, have the potential to overcome concerns about FP and increase satisfaction and continuation rates [2, 3].

Based on national-level data, unmet need for modern contraceptive methods was high among all women in Côte d’Ivoire, at 17% in December 2022, and contraceptive prevalence was low, at only 27% [4]. Among those currently using modern methods, 62% stated that they did not receive information on side effects for their method, and only 23% reported receiving comprehensive counseling on contraceptive methods [4]. Recent qualitative research in Abidjan suggested barriers to contraceptive use for both women and men included fear of side effects and misconceptions, such as the idea that FP leads to infertility [5].

Increasing uptake of FP services among women and couples who would benefit will require addressing a multitude of issues pertaining to both service delivery and service demand. This includes providing access to accurate and complete information on FP and contraceptive methods to those who need it. There is keen interest in the potential role of digital approaches to providing FP information, such as through social media, to address barriers to accessing and using contraceptives [1]. While there is a growing body of literature on mobile-phone-based SRH interventions, much of the focus historically has been on one-way or two-way mobile messaging programs [6] and on the use of text messages to increase knowledge or disseminate information, often with little or no individual tailoring [7–9]. However, in recent years, there has been a shift toward newer technologies like smartphone apps, social network sites or chatbots. Chatbots relying on artificial intelligence (AI) are also increasingly being used to support more interactive and tailored communication strategies [6, 10]. While some one-way communication initiatives have been shown to improve SRH knowledge, a limited but growing body of evidence suggests that interactive and tailored communication strategies are associated with greater effects on a variety of health-related outcomes [11]. Comprehensive data on these newer strategies is still emerging, and evidence of their application to FP remains relatively limited.

The feasibility of using mobile technologies to provide access to FP information is rapidly increasing. In 2021, 67% of the world population had mobile services, and over half (53%) of the global population was connected to mobile internet [12]. In 2022, the cellular penetration was at 164.5% in Côte d’Ivoire, meaning that a significant proportion of the population has more than one phone [13]. However, men in Côte d’Ivoire have a higher rate of mobile phone ownership than women (90% vs. 82%), a gap that is wider in rural areas compared to urban ones (12% vs. 6%). This gender inequity is even more significant when it comes to the use of mobile internet; 52% of men in Côte d’Ivoire report access to mobile internet, compared to only 28% of women [14]. Approximately 36.3% (9.9 million) of the population had access to the internet, and there were an estimated 6.4 million social media users in 2022 [15]. Facebook was by far the most popular social media application with an estimated 5.7 million users, ~37.2% of whom were female [15]. The number of social media users in the country is increasing rapidly, by 20% from 2020 to 2021 and 8.5% from 2021 and 2022 [15, 16].

Building on this small, but growing body of literature, this study aimed to assess the feasibility^1^ and acceptability of using an interactive, online and health-promotion chatbot designed to improve FP knowledge and access among adolescents and young people in Côte d’Ivoire with internet access. Specifically, this study aimed to:

(1) Describe user characteristics in terms of both who is using the tool (demographics) and why individuals of both sexes choose to use the tool: how they learned of it, what attracted their attention and their motivations for accessing it.
(2) Describe how users engage with the tool, including: which areas of the chatbot are accessed and by whom; what trajectories users take through the chatbot (including where and why users abandon the tool) and perceptions of the tool in terms of usability, content and relevance.
(3) Document users’ perceptions of the tool’s effect on their FP-related self-efficacy and behavioral intentions and explore female users’ perceptions of the effect on their subsequent FP-related actions.
(4) Document the interest of female tool users in other forms of self-care related to the tool’s content.

## METHODS

### Study design

We conducted a cross-sectional, descriptive and mixed-methods study with women and men in Côte d’Ivoire who interacted with the intervention from 11 January 2022 to 15 July 2022. The study collected data through three sources: structured online questionnaires (one for women, one for men); in-depth interviews (IDIs) conducted by telephone with a purposely selected subgroup of female chatbot users, and de-identified data extracted for the study from the chatbot back-end database. The study was reviewed and approved by the FHI 360 Protection of Human Subjects Committee in the U.S. and the Côte d’Ivoire National Ethics Committee for Life Sciences and Health.

### Intervention description

The Counseling for Choice (C4C) chatbot described in this study is promoted in Côte d’Ivoire using the name ‘l’Assistante de Gabi’ (Gabi’s Assistant), referencing an avatar, Gabi, already used in other digital interventions by the implementing organization (Supplemental Materials 1 and 2). It is available to anyone through Meta’s Messenger and WhatsApp applications, and it has been marketed to women in Côte d’Ivoire through social media advertisements and ‘influencers’ since 2020 (Supplemental Materials 3). In addition to sections focused on general information about contraceptive methods and menstrual health, the chatbot has a section modeled after the C4C approach to contraceptive counseling. Developed by Population Services International (PSI), C4C is an evidence-based, client-centered approach to FP counseling. It tailors counseling sessions to each client, recognizing that based on personal preference or past experience, clients attach different value to the various advantages and disadvantages linked with each FP method [18]. The C4C chatbot translates the principles of in-person, provider-led C4C counseling into the intervention’s digital, client-facing tool.

Once a user accesses the chatbot through a link, the person is introduced to ‘Gabi’s Assistant’, the chatbot’s persona or avatar. ‘Gabi’s Assistant’ begins by asking the user if s/he is a woman or a man, her/his age group, if s/he resides within or outside of Abidjan and what information s/he is seeking. The chatbot has three ‘branches’ or sections with specific information. The first two sections, available to both women and men, are informative and include: (i) ‘All Methods’, which contains information on all available modern FP methods and (ii) ‘Menstruation’, which contains information on the menstrual cycle. The third branch, ‘Recommended Methods’, operationalizes the C4C decision-making approach, resulting in an individualized chat with ‘Gabi’s Assistant’ that tailors recommendations to specific concerns the user selects. As this portion of the chatbot (referred to hereafter as the ‘Recommended Methods’ section) asks about specific concerns that an FP method user may have or experience, it is only available to users who self-report to be women. Information on Côte d’Ivoire’s commonly available modern FP methods was included in the chatbot: oral contraceptive pills, injectables,^2^ Cycle Beads, copper intra-uterine device (IUDs), implants, female sterilization, male sterilization, male condoms and emergency contraceptive pills (ECPs).

In the ‘Recommended Methods’ section, the chatbot asks the user to select the FP method benefits that are most important to her. These benefits include a range of method characteristics, including effectiveness at preventing pregnancy, quick return to fertility, self-administration, and predictable periods, among others [19]. Then, the tool uses this information to suggest the two to three FP methods that most closely correspond to the user’s responses, as a provider using the C4C approach would do during an in-person counseling session. After viewing information about FP methods in either the ‘Recommended Methods’ or ‘All Methods’ sections, women self-reporting to be in the Abidjan region are then offered a referral code (QR code) to a clinic supported by PSI/Côte d’Ivoire. With the code, these clinics provide initial FP services free-of-charge to the client.^3^ The chatbot program monitoring database tracks data for each user account that accesses the chatbot and the responses that were selected for each question. Referrals and referral completion are tracked in the database for users who go to a PSI clinic using the QR code. Users are also able to self-report a completed referral within the chatbot.

### Sampling

#### Structured online survey

We recruited a convenience sample of all adult chatbot users who agreed to participate in a survey about their experiences with the chatbot during the six-month study period, during which PSI generated demand for the chatbot via existing social media channels.^4^ We aimed to recruit participants until we reached a maximum of 300 survey responses or reached the end of the data collection period, whichever came first. We chose to cap recruitment at 300 surveys because we believed 300 would be sufficient to capture a range of potential user characteristics and perspectives in the available time frame, based on historical chatbot use data. Participants were recruited online through a survey link coded directly into the Gabi chatbot. Chatbot users who reached the referral prompt portion of the ‘Recommended Methods’ or ‘All Methods’ branches (e.g. received an FP service referral code or declined to receive such a code) or elected to go back to an earlier section of the chatbot (before reaching the referral prompt) were shown a survey invitation message and survey link within the chatbot itself. Eligible participants were provided information on the study, its risks and potential benefits, and a summary of the data to be collected, and were asked to provide consent to take part in the study, after which they completed a short, self-directed, structured online questionnaire.

The survey covered user socio-demographics, mobile device ownership and use, reasons for accessing the chatbot, perspectives on chatbot content, as well as barriers or challenges encountered using the chatbot. Additionally, among female respondents only, we collected data on the perceived effect of the chatbot on FP-related self-efficacy and on contraceptive-related behavioral intentions.

#### In-depth interviews

We recruited three sub-groups of female chatbot users for IDIs: users who interacted only with the informational components of the chatbot; users who went to the ‘Recommended Methods’ section but did not complete it; and users who interacted with this section and were provided with a list of 2–3 recommended methods. We aimed to conduct up to 36 IDIs, approximately equally distributed among the three sub-groups. Participants were selected from among those who agreed to provide contact information to take part in the IDIs after completing the online survey. Contact information was recorded in a separate database with no link to respondents’ questionnaire data. Potential participants self-reported their interactions with the chatbot during screening to determine eligibility and ensure sub-group quotas were met.

IDIs were used to explore participants’ experiences with the chatbot tool in greater detail, including any difficulties encountered during interaction. We also asked for suggestions to improve or change the tool to better meet the respondent’s informational needs, including perceptions of the ‘Recommended Methods’ section. In addition, we explored reasons for accepting a referral or not (if relevant), and whether respondents sought care or services, and why.

#### Back-end data

Those responsible for implementing and monitoring the chatbot intervention extracted and de-identified chatbot back-end data from the program database. These data on users who accessed the chatbot during the study period included: user age, gender and geographic location (Abidjan/outside Abidjan); information about the user’s interactions with the tool, including number of interactions with the tool during the study period, and for each interaction, which of the three sections the user explored (all methods, menstruation and recommended methods); time spent interacting with the tool; and whether the user accepted a referral code.

#### Data analysis

Quantitative data from the structured questionnaire and from the chatbot database were descriptively analyzed, reporting measures of central tendency, such as means/medians, spread (range) and proportions depending on variable type (e.g. continuous, discrete and categorical). Simple, descriptive cross-tabulations and stratified tables are presented to examine the distribution of variables by sub-groups. Qualitative data from IDIs were transcribed and analyzed in the original French. A codebook was developed based on key concepts related to the study objectives and was modified through immersive reading of the first few transcripts to add emergent codes. QSR NVivo software was used to code data and conduct a thematic content analysis. Data reduction techniques were used to examine codes in detail for sub-themes and patterns across the IDIs. Summary reports were developed and translated into English.

## RESULTS

### Back-end chatbot data

During the study period between 13 January - 11 July 2022, a total of 2081 unique individuals used the chatbot (1722 one-time users and 359 repeat users) based on back-end monitoring data collected by the chatbot. These users clicked on the chatbot a total of 4623 times (Table 1). Most of the unique individuals who used the chatbot identified as female (84%). Those aged 19–24 years accounted for the majority of the chatbot users (60%). Over two-thirds (68%) of chatbot users reported living in the capital city of Abidjan. A total of 959 referrals were issued by the chatbot during this period, of which 84 (9%) had been redeemed (Supplemental Materials 4).

**Table 1:** Characteristics of chatbot users from chatbot back-end data

Variable		Unique users (*N* = 2081)	%
**Gender**			
	Female	1756	84
	Male	325	16
**Age**			
	<19 years	299	14
	19–24 years	1250	60
	25+ years	532	26
**City**			
	Abidjan	1420	68
	Outside Abidjan	661	32
**Chatbot journey (unique users)**			
	Clicked on a branch	1752	84
	Engaged with one of the 3 branches^*^	1681	81
	Completed the branch^*^^*^	1111	53
**Referrals/vouchers**			
	Referrals issued	959	46
	Redeemed referrals	84	4
**Branch views (clicks)**		(4623 clicks)	
	All methods	2783	60
	Recommended methods	922	20
	Period Information	918	20
**Study survey**			
	Clicked on survey link	399	24^*^^*^^*^
	Eligible for study and consented to participate	284	71
	Completed study survey	280	99

^*^Engagement was defined as a user clicking on content within one or more of the three branches.

^**^For the ‘All Methods’ and ‘Period Information’ branches, reaching the ‘end’ of a branch was defined as following the sequence of prompts until no further new information on the originally selected topic was available (users could navigate through multiple topics, if desired). For the ‘Recommended Methods’ branch, completing the branch was defined as receiving a set of recommended methods.

^***^Proportion of unique users who engaged with one of the three branches who also clicked on the study survey link.

The remaining data presented were obtained prospectively during the e-survey and IDIs.

### Participant characteristics

A total of 280 men and women took part in the study (Table 2). Most respondents were female (96%), had university/technical education (159/262, 61%) and resided in the capital city of Abidjan (187/262, 71%). Participants were young in general, with an average age of 22 years. Just over half of the respondents (141/261, 54%) reported being single/not in a relationship and nearly half (118/246, 48%) reported currently using an FP method. The most reported current FP methods were male condoms (51/118, 43%), followed by contraceptive implants (23/118, 19%) and oral contraceptive pills (OCPs) (23/118, 19%). Approximately 19% (23/118) of respondents reported currently using ECPs as their main contraceptive method.

**Table 2:** Participant characteristics (*n* = 280)

**Variable**	**Total (*N* = 280)**	
**Age (Mean, SD)**	22	4
	**N**	**%**
**Female**	268	96
**Highest level of education** (*n* = 262)		
Primary or less	7	2
Secondary	87	33
University/technical	159	61
Other	9	3
**Paid work** (*n* = 260)	62	24
**Marital status** (*n* = 261)		
Living with partner	44	17
In relationship but not cohabitating	76	29
Single/not in a relationship	141	54
**Reside in Abidjan** (*n* = 262)	187	71
**Ever been pregnant** (*n* = 251)	115	46
**Ever used FP** (*n* = 262)	181	69
**Currently using FP** (*n* = 246)	118	48
**Current method used** (*n* = 118)		
Female sterilization	1	0.8
Male sterilization	4	3
Implant	23	19
Injectable	19	16
IUD	0	0
Oral contraceptive pills	23	19
Emergency contraceptive pills	23	19
Male condoms	51	43
Female condoms	2	2
Spermicide	1	0.8
Fertility awareness methods	8	7
Diaphragm	0	0
Withdrawal	17	14
Other method	2	2

In total, 28 female respondents completed IDIs. Across the three sub-groups, we recruited 12 users who interacted with the informational components of the chatbot, 6 users who interacted with but did not complete the ‘Recommended Methods Section’ and 10 users who received a list of recommended methods.

### Social media and chatbot use

Nearly all survey respondents (98%) reported using a telephone/tablet for social media, the majority of which (98%) were owned by the respondent themselves (Table 3). Most respondents (81%) reported daily social media use, with platforms such as Facebook (90%), WhatsApp (97%), TikTok (48%), and YouTube (47%) being among the most commonly visited.

**Table 3:** Social media, device, and chatbot use

**Variable**	**Total (*N* = 280)**	
	***N***	%
**Device used for social media** (*n* = 256)		
Telephone/tablet	252	98
Laptop	3	1
Other	1	0.4
**Who owns this device?** (*n* = 255)		
Self	250	98
Someone else	5	2
**Share device with someone else** (*n* = 255)	28	11
**Social media platforms used** (*n* = 257)		
Facebook	232	90
WhatsApp	249	97
Twitter	19	7
Pinterest	24	9
YouTube	121	47
Instagram	99	39
LinkedIn	22	9
TikTok	124	48
**Frequency of social media use** (*n* = 255)		
Every day	207	81
A few times a week	48	19
**How learned about chatbot** (*n* = 240)		
Facebook	216	90
Instagram	6	3
WhatsApp	8	3
Friend, relative or partner	7	3
Other	3	1
**Number of times visited chatbot** (*n* = 241)		
Once	205	85
Twice	21	9
Three or more times	15	6
**Branch(es) visited** ^*^		
Method recommendations	176	63
Method information	63	23
Period information	83	30

^*^The chatbot branches visited sum to more than 280, since survey respondents could have visited one or more branches.

Most respondents reported originally learning about the Gabi chatbot via Facebook (90%) and having used the Gabi chatbot just once prior to taking the survey (85%), while 6% reported having accessed the chatbot three or more times. Among survey respondents, the ‘Recommended Methods’ branch was the most frequently visited (*n* = 176, 63%).

### Reasons for using the chatbot

The most commonly reported reasons for using the chatbot included to ‘obtain information for myself’ (73%) or because a respondent was ‘just curious’ (36%) (Table 4). Respondents expressing interest in period-related information did so primarily because of a self-reported desire to better understand the menstrual cycle (57%), or because they were experiencing irregular periods (26%). Respondents interested in FP-related information accessed the ‘All Methods’ portion of the chatbot mainly because they intended to start using an FP method (47%) or wanted to learn more about the method they were currently using (24%), though nearly 20% reported accessing this branch out of general curiosity.

**Table 4:** Reasons for using chatbot and chatbot components among survey respondents

**Variable**	**Total (*N* = 280)**	
	***N***	%
**Why did you use the chatbot** (*n* = 280)^*^		
Information for myself	203	73
Just curious	100	36
Information for someone else	5	2
Used it with someone else	1	0.4
Other reason	8	3
**Why did you want to learn about periods?** (*n* = 82)		
To better understand menstrual cycle	47	57
I have irregular periods or menstrual problems	21	26
Just curious	13	16
Other	1	1
**Why did you want to learn more about FP methods** (*N* = 62)		
I plan to use a FP method	29	47
I want to learn more about the method I/my partner is currently using	15	24
Just curious	12	19
I want to choose a FP method to start using	5	8
I plan to change method	1	2
Other	0	0

^*^Respondents could select more than option for this question.

The qualitative interviews explored in more detail the reasons why respondents were seeking information about FP or periods from the chatbot. Reasons for accessing the chatbot included wanting to have a better idea of the contraceptive methods available in Cote d’Ivoire, looking for information to guide method selection (e.g. advantages and disadvantages of different methods), or seeking additional information on the contraceptive method already being used (e.g. side effects, effectiveness, impact on the menstrual cycle). One user told us, ‘I was looking for a contraceptive method and I didn't know which one to take and I saw this as an opportunity to get all the information that would allow me to change my mind’ (22-year-old woman).

Several IDI participants also reported accessing the chatbot to verify information they had previously heard about contraceptives, but that they were not fully confident about. One young woman said:

‘These are the implants that I wanted to use, but I was told no because I am overweight. And for people who are overweight, it can cause problems, it can be lost and blend into the skin. So, I decided to go there to see if it's really true. That's what people were telling me, but I saw that it wasn’t.’ (19-year-old woman).

For those who were interested in information on menstruation, several women reported that they were looking for information on how to calculate the menstrual cycle, how to determine if a cycle is regular or irregular and how to manage menstrual pain.

### Perceptions of the chatbot

Nearly all IDI respondents felt the chatbot was user-friendly, both in terms of the quality of information presented and its structure. One respondent commented:

‘Yes, I really liked it because they don’t give us all the information at once, they give us the explanations first and we choose. They talk about common methods and when you click on them, the answers come as you go along. It’s easy to understand.’ (22-year-old woman)

Users also expressed appreciation for the automation of responses in the chatbot, the speed of Gabi’s responses, the attractiveness of the chatbot interface, the fluidity and good structuring of the chatbot’s algorithm and Gabi’s ‘friendly and polite nature.’ One respondent explained, ‘Let's say that I had the impression that I was talking with someone who was in front of me. I was talking and the person answered me as if he knew me’ (21-year-old woman).

However, some IDI respondents did report experiencing challenges in understanding or answering some of the questions posed by the chatbot. Several respondents expressed concerns that other (sometimes hypothetical) users, especially those without a high level of education, would have difficulties understanding some of the expressions used by Gabi, especially medical terms or the more formal French language used by the chatbot. One respondent said:

‘You didn't use down-to-earth words, so that's going to be a problem. The French used is very complicated. Some words were hard for me to understand, so I had to resort to the French dictionary*.*’ (29-year-old woman)

#### Perceptions of the recommended methods section

Among those survey respondents who visited the ‘Recommended Methods’ branch, over 88% found it very easy or easy enough to answer Gabi’s questions (Table 5). The most common challenges reported with this section included feeling that there were too many questions (28%) or that the questions were hard to understand or to answer (27%). Only 9% of participants reported experiencing any technical difficulties when interacting with this section.

**Table 5:** Perceptions of the chatbot’s ‘Recommended Methods’ section among e-survey respondents

**Variable**	**Total (*N* = 280)**	
	***N***	%
**How easy/difficult was it to answer Gabi's questions?**		
Difficult	15	12
Easy enough	44	35
Very easy	68	54
**How easy/difficult was it to use the Recommended Methods section?**		
Very difficult	1	0.8
A bit difficult	17	13
Easy enough	41	32
Very easy	68	54
**Challenges with Recommended Methods Section**		
Questions were hard to understand or answer	48	27
There were too many questions	49	28
I had technical difficulties	16	9
Other	23	13

#### Perception of the chatbot content

**Figure 1:**
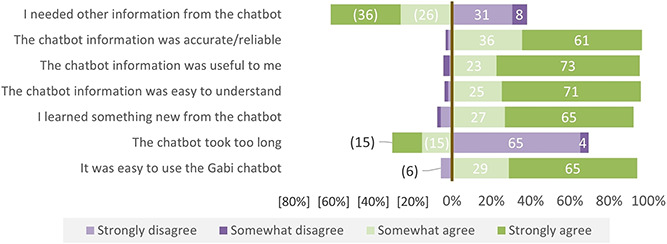
How much do you agree with the following statements about the chatbot? (*N* = 159). ^*^All positive responses are indicated to the right of the axis, and all negative responses are shown on to the left. The axis is set at 0%, with positive responses ranging from 0 to 100% on the right-hand side, and negative responses ranging from [0 to 100%] on the left-hand side. For positively framed questions (e.g. ‘The chatbot information was useful to me’), the options for ‘somewhat agree’ and ‘strongly agree’ reflect positive sentiments and are shown to the right of the axis. Because some questions (e.g. ‘the chatbot took too long’) were framed in the negative, rather than the positive, the response options for these negatively framed questions have been flipped. As a result, the ‘strongly disagree’ and ‘somewhat disagree’ options (reflecting positive views of the chatbot) appear to the right of the axis. This allows one to compare the strength of positive (and negative) sentiments across questions, regardless of the way they were framed to the survey respondent

Over 90% of survey respondents agreed (27%) or strongly agreed (65%) that they learned something new from the chatbot (Fig. 1). Nearly all survey participants agreed or strongly agreed that the chatbot information was useful to them (96%), that the information was easy to understand (96%) and that the information seemed accurate and reliable (97%).

However, more than half of survey participants agreed (26%) or strongly agreed (34%) that they needed more information from the chatbot than what they were able to find during their interaction. This finding was supported by some IDIs, identifying additional information needs for specific FP methods. One woman told us:

‘It's about the morning-after pill, that's where I said there was missing information because it was talking about the progesterone-based pills, the 3-day pills. There was no information about the 5-day pills, no information that said if you take a 3-day pill after fertilization, it has no effect.’ (22-year-old woman)

Another user complained that the chatbot was unable to answer method-specific follow-up questions that she had, such as the difference between the different types of injectables, including self-injection options, saying:

‘... the questions I asked were not answered. Because they said that injectables could be self-injected, so I asked how, since I don't have the time to go to the hospital all the time. But I didn't have an answer.’ (26-year-old woman)

Participants also desired more information on the specific side effects of each contraceptive method, which they felt would reassure potential users and address doubts, rumors, misconceptions or misinformation circulating about contraceptive methods. One interviewee commented:

‘People have a habit of saying a lot of things about contraceptives that aren't necessarily encouraging. So, I would like to have questions where those kinds of things are denied or clearly explained.’ (28-year-old woman)

Another participant mentioned not finding information on less commonly available contraceptive methods in the chatbot, such as patches and spermicides. One user reported, ‘As I said there, there is a contraceptive method that I see in the movies that is called, I believe, the patch that is stuck on the body… Information on patches, for example, spermicides, is not in there, or I might have missed it if it is in there.’ (28-year-old woman)

Users were also interested in additional sexual and reproductive health topics, including understanding more about the causes and solutions to irregularities in the menstrual cycle, managing pain during periods, management of their sexual and reproductive health, preventing, diagnosing and treating sexually transmitted infections, and managing pregnancy and the postpartum period.

#### Perceived effect of the chatbot on self-efficacy and behavioral intentions

**Figure 2:**
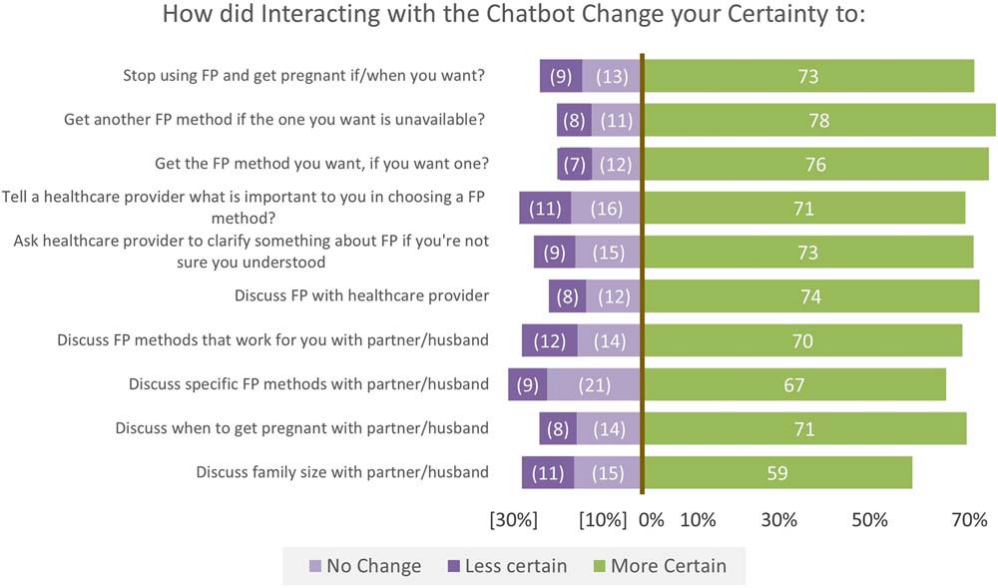
Perceived effects of the chatbot on self-efficacy and behavioral intentions. ^*^To facilitate comparison across questions, all positive response options (e.g. ‘more certain’) are indicated to the right of the axis at 0%, and all negative or neutral responses (e.g. ‘no change’ or ‘less certain’) are indicated to the left of the axis. This allows the reader to quickly observe which questions had the strongest positive (or negative) sentiment at a glance

After using the chatbot, survey respondents reported feeling more confident about engaging in FP-related discussions and actions (Fig. 2). After interacting with the chatbot, 78% felt more certain that they could get another FP method if the one that they want was not available, and 74% felt more certain that they could discuss FP with a healthcare provider. Relatively fewer respondents reported feeling more certain about discussing family size with a partner/husband after interacting with the chatbot, though more than half of respondents (59%) reported feeling more confident about engaging on this topic.

The increased confidence in FP-related discussions/actions following chatbot use was also observed in the qualitative data. Most IDI respondents (25/28) highlighted the influence of the chatbot on their ability to discuss FP with other people. Participants reported feeling more confident discussing FP because they felt better informed following their interaction with the chatbot, including having a better understanding of specific FP methods, how to use them, and their relative advantages or disadvantages. Women also reported feeling more able to support their own FP choices and, if necessary, to push back against skeptical providers or partners with differing opinions. One interviewee explained that:

“Yes. I can do it. I can do it. Because, when I went to the hospital, there I was doing the injectable. When…I left for my next appointment there, I told the midwife that I wanted to stop the injectable. She told me ‘Why?’ I told her that ‘When I came to do [the injection], they didn’t tell me this, they didn’t tell me that, so I want to change my method.’ I understood thanks to Gabi that it was the injectable that cut my periods, so I told her, ‘Here, I want to change because it cuts my periods. I can’t see my periods anymore.’” (26-year-old woman)

Interviewees also reported feeling more confident to discuss contraceptives with partners or spouses after interacting with Gabi. Fifteen IDI participants reported having such discussions with their partners after using the chatbot. One woman told us:

‘I said to him, “This is what I learned. Since we’re not ready for a pregnancy, why not use birth control? Now it’s up to me to tell him that there are pills, injections, and implants... there are condoms too, so I would prefer that to avoid pregnancy, we have to use a method.”’ (24-year-old woman)

In addition, most IDI participants (23/28) reported a positive influence of the chatbot on their intention to use or change contraceptive methods. For these women, the lack of information on the methods available, the fear of prejudices shared by those around them and misconceptions about FP were the main obstacles to adopting a contraceptive method. The information provided by the chatbot helped to reassure them. Specifically, women said they trusted the chatbot because it clearly explained all the disadvantages of the methods, as opposed to only the advantages, and addressed specific user questions, including misperceptions related to method side effects.

#### Actions/changes after interacting with the chatbot

Most (88%) survey respondents reported receiving a list of recommended contraceptive methods after interacting with the chatbot. Of these, however, only 41 (37%) opted to receive a referral code to receive services from an FP provider. At the time of the survey, only 12% of women self-reported (*n* = 5) having redeemed their referral code, though 66% (*n* = 27) planned to in the future. Among IDI participants (all of whose interviews took place after the survey), a total of ten women reported having adopted or changed contraceptive methods after interacting with the chatbot. Specifically, five adopted a method (pills and implants) and five others changed methods (e.g. from pills or condoms to implants or injectables). These changes were reportedly motivated by the information received from the chatbot on the use, duration of action, advantages and disadvantages of each contraceptive method. One respondent reported:

‘I wanted to change but I didn't know which method to take. I went through Gabi and saw the pros and cons of each method and decided to take the injectables, I didn't want to take the implant because of the length of time. I was told that there was a 3-year and 5-year implants. I wanted a method that could last but not more than a year. When I went to Gabi, it was because of the length of time, the side effects and the return to fertility, I found that the injectable was better. I left the pill because of the forgetfulness.’ (27-year-old woman)

### Suggestion for improving the chatbot

During the IDIs, respondents suggested changes to the chatbot content and structure to improve the user experience. These changes included the addition of audio (for those with lower literacy or vision-related disabilities), expanding the content to include a broader range of sexual and reproductive health content (such as pregnancy and STIs), an option for users to ask open-ended questions or to post free-text responses to Gabi’s questions (e.g. if the response options provided by the chatbot don’t apply), a frequently asked questions section to address other common issues not currently covered in the chatbot, adding a hotline or phone-contact for live support, and a list of midwives, gynecologists or other service providers with contact information. IDI respondents also suggested using simpler, colloquial language to make the chatbot more understandable (especially for those with lower levels of education), making the chatbot available in an app format, reducing text length and adding more images, videos, colors, emojis, etc., adding a chatbot user guide, expanding the WhatsApp chatbot features, including the use of voice notes (especially beneficial for those with low literacy levels), and adding the possibility of offline use.

## DISCUSSION

This mixed-methods study found the C4C chatbot to be feasible to implement and highly acceptable among study participants in Cote d’Ivoire. Participants indicated that interaction with the chatbot increased their confidence to engage in FP-related discussions and actions, such as communicating with a sexual partner about method uptake or talking to a provider about switching to a preferred method. Survey and IDI participants generally found the information provided by the chatbot to be relevant, trustworthy and easy to understand. Previous research on the development of health-related chatbots has highlighted the importance of trust in engaging users and in providing information that is seen as reliable and actionable to users. Elements that were prioritized by users elsewhere included clear branding, and perceived emotional sensitivity and empathy on the part of the chatbot [20].

Other systematic reviews on the use of AI, including chatbots and conversational agents, in healthcare in low- and middle-income countries have found mixed results, with challenges identified around user-friendliness, adaptation to local contexts and reliability issues [10, 21]. Participants in our study felt that the chatbot was easy to use, and most reported not encountering any problems (such as connection challenges) during their session. However, IDI participants expressed concerns around the chatbot language, including that the level of French used by the chatbot may be difficult for some users to understand. While translation into more local languages may be costly, our findings suggest that this may improve the user experience and understanding, especially for users with lower levels of education.

The commonest issue reported by respondents in our study was a desire for additional information that they didn’t find from the chatbot, and many of the suggestions for ways to improve the chatbot included strategies for more personalized follow-up or in-depth questions that the algorithm-based chatbot was not capable of answering. Continued advancements in AI may allow for more responsiveness on the part of the chatbot, including the ability for users to ask open-ended questions and explore a broader range of reproductive health topics. However, this is an area where more research is needed, including around the clinical, legal and ethical implications of AI chatbots, especially those using generative models, for health. While studies have shown a value for chatbots in addressing simple, easily automated or repetitive tasks (such as sorting and triaging customer service calls), data on their effectiveness for addressing more complex, open-ended tasks related to the promotion of health or the treatment and prevention of disease is less firmly established [22]. Further data are also needed on SRH chatbots’ cost-effectiveness, their privacy and security implications and effective strategies for linking chatbot users to the wider health system [23].

Our study suggests that while participants self-reported feeling more confident about discussions related to FP following engagement with the chatbot, relatively few (41/280) opted to receive a referral for services, and only a minority (5/280) of those surveyed had completed a referral and received FP services at the time of the survey. However, since surveys were completed within hours to days of interacting with a chatbot, most respondents had not had time to complete the referral before the survey. Importantly, roughly 2/3 who had received a referral said they intended to use it in the future. Electronic referrals are a common feature of many digital FP tools, included as a component in 36 of 102 such tools in a recent landscaping report [24]. Though evidence around the efficiency and effectiveness of digital referrals is emergent, a growing body of research suggests that though these models may have higher start-up costs relative to other referral systems, they can be effective and are being increasingly rolled out [25].

Though referral uptake was relatively low at the time of the survey, it is possible that chatbot engagement resulted in other, more upstream benefits to users, such as increased awareness of FP, increased FP-related knowledge or increased confidence/empowerment. These outcomes are important and may result in increased FP uptake over a longer time horizon than observed in our study. For digital tools, especially those targeting adolescents and young people who may not yet be sexually active or currently in need of FP, these outcomes are especially relevant. Improved understanding of the pathways to FP use, the role of digital interventions on journeys to FP, and appropriate measures of effectiveness for digital tools are still needed. Women and girls in our study reported in both the survey and IDIs that they felt more confident in engaging in FP-related discussions and actions following their interaction with the chatbot and provided examples of conversations with partners, providers and even method uptake or switching following their engagement with the chatbot. While this study is descriptive and did not have a control group to enable estimation of chatbot effectiveness, these findings suggest future research may be useful to quantify the size of the chatbot effects on empowerment, user confidence, and FP-related actions.

Finally, our survey respondents were overwhelmingly female and tended to be urban singles between the ages of 19–24 years with a university education. While this mirrors the user base of the chatbot more broadly, these women are not representative of the wider Ivorian population. By 2021, only 32% of Ivorian women had a secondary education or above [26], and just under half of the population resides in rural areas [27]. As with other digital interventions for FP and reproductive health, the reach of the chatbot into more affluent, urban, better-educated populations raises concerns about equity [28]. This serves as a reminder that digital interventions should not be scaled as stand-alone interventions but should be seen as part of the broader health system and well-integrated into larger health programs that utilize a mix of approaches (including potentially more intensive and costly in-person interventions) to reach population segments with more limited access to digital tools, lower digital literacy, or other barriers.

## LIMITATIONS

This study is subject to some important limitations. Most importantly, the study recruited a convenience sample of participants; therefore, the extent to which findings can be generalized to the larger target population is unknown. Further, traffic was driven to the chatbot through demand creation activities during the recruitment period (which was extended from 3 to 6 months because of delays in reaching the intended sample size), which may have resulted in a different sample than one obtained without demand creation efforts. Additionally, the self-reported effects of the chatbot should only be considered from the perspectives of the participants and not an objective assessment of intervention effects. This is especially true given the cross-sectional nature of the study, our lack of a control or comparison group and the fact that the extent of participant’s chatbot interactions was based on self-report, rather than more objective measures. Further, it is possible that social desirability bias resulted in more positive assessments of the chatbots’ influence and impact, though we attempted to limit this by assessing perceived chatbot effects via a self-directed online survey instrument. Future research may be needed to estimate the effectiveness of the chatbot on intermediate outcomes, such as self-efficacy and empowerment, and the role that factors like digital literacy and trust play in mediating the impact of the chatbot on FP-related outcomes. Future research should also explore the impacts of digital interventions like chatbots on real-world behavior, including referral completion and FP uptake.

## CONCLUSION

This study adds to the growing literature on the impacts of digital interventions for FP and sexual and reproductive health in low- and middle-income countries. This is one of the few studies examining the feasibility and acceptability of an informational chatbot targeting adolescents and young people [29] in a West African setting. Our findings suggest that the intervention was both feasible to implement and acceptable to participants. Future research should examine the effectiveness of digital interventions, such as the C4C chatbot, on health-related outcomes, such as self-efficacy to seek and access FP services and to obtain FP methods of choice. Implementers and researchers should also continue to explore the reach of these digital tools, and the implications of digital interventions for outcomes related to FP equity.

## Supplementary Material

Supplemental_Materials_1_to_4_oqae027

## Data Availability

The data underlying this article are available in Harvard Dataverse at: https://dataverse.harvard.edu/dataset.xhtml?persistentId=doi:10.7910/DVN/8O7EWH.

## Footnotes

1 We defined feasibility as the ‘Actual fit or utility’ or ‘suitability’ of the chatbot ‘for everyday use; practicability.’ Feasibility was determined based on the target audience’s use/uptake of the chatbot, as well as user feedback on the relevance and utility of the information provided and the usability of the tool [17].

2 Both DMPA-IM and DMPA-SC are available in Côte d’Ivoire.

3 Women who do not reside in Abidjan are not provided a referral code because there are no linked PSI-supported clinics outside of Abidjan; however, all users are informed that they can receive services at any FP clinic near them. FP services are free-of-charge to women 24 years and under in Côte d’Ivoire, but women 25 years and older can be charged for the service.

4 Originally the study planned to recruit participants for 3 months, but this was later extended to 6 months due to low enrollment during the initial 3-month period. During this period PSI conducted two rounds of social media demand creation to drive traffic to the chatbot. These Facebook-based campaigns ran from December to April 2022 and again from June to July 2022 and cost $8500 in total. Chatbot traffic increased by 31% (from 7158 unique views to 9403) during the study period compared to the period preceding the campaign’s launch, for an incremental cost of $3.79 per additional unique chatbot user. Those viewing the campaign were provided with information about, and a link to, the chatbot, but were not compensated or otherwise incentivized to access or interact with the chatbot.
