# Supplementary material for: Mixed-methods, cross-sectional assessment of a client-facing, family planning counseling chatbot in Côte d’Ivoire
Source: Oxf Open Digit Health. 2024 Aug 3;2:oqae027. doi: 10.1093/oodh/oqae027 (PMC11932393; doi:10.1093/oodh/oqae027)

# Title: Mixed-Methods, Cross-sectional Assessment of a Client-facing, Family Planning Counseling Chatbot in Côte d’Ivoire

**Authors:** Kristen M. Little^1*^, Ndola Prata^2^, Kpebo Djoukou Olga Denise^3^, Nadia Tefouet^4^, Jean Christophe Fotso^4^, Alexandra Angel^5^, Sarah Brittingham^6^, Paul Bouanchaud^1^, Amadou Donapoho Soro^7^, Lisa Dulli^8^

^1^ Strategy & Insights Department, Population Services International (PSI), Washington, DC, USA

^2^ Reproductive, Maternal, Newborn, Child and Adolescent Health (RMNCAH), Evidence for Sustainable Human Development (EVIHDAF), Yaounde, Cameroon and the School of Public Health, University of California Berkeley, Berkeley, California, USA

^3^ Unit of Research in Maternal and Child Health, National Institute of Public Health, Abidjan, Côte d'Ivoire

^4^ RMNCAH, EVIHDAF, Yaounde, Cameroon

^5^Sexual and Reproductive Health Department, PSI, Washington, DC, USA

^6^ Scientific, Technical and Evidence Advancement Department, FHI 360, Durham, NC, USA

^7^ Strategic Information Department, PSI/Côte d’Ivoire, Abidjan, Côte d’Ivoire

^8^ Global Health and Population Research Department, FHI 360, Durham, NC, USA

**Corresponding Author:**

^*^Kristen Little

Strategy & Insights Department

Population Services International

1120 19^th^ Street NW, Suite 600

Washington, DC 20036

[klittle@psi.org](mailto:klittle@psi.org)

**Supplemental Materials 1:** Gabi the virtual assistant


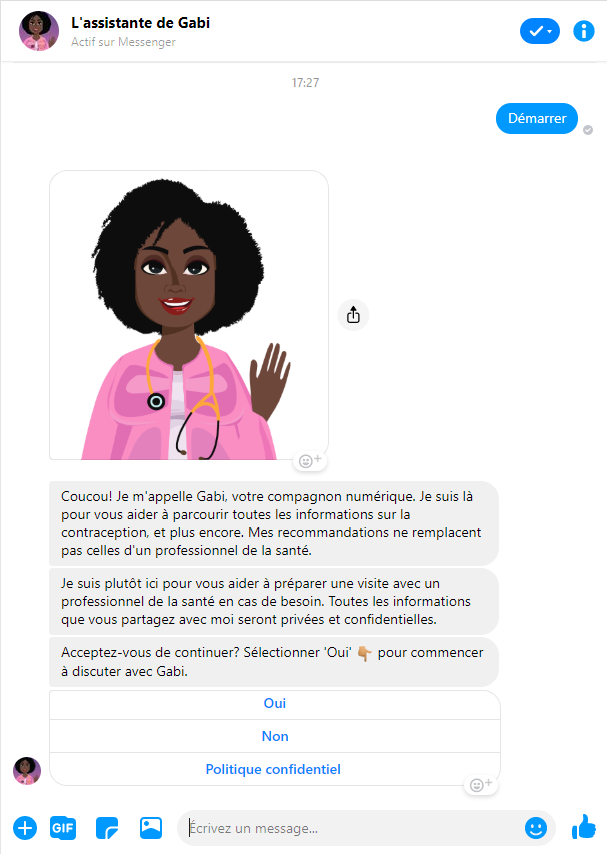


**Supplemental Materials 2.** Gabi’s introductory questions

**
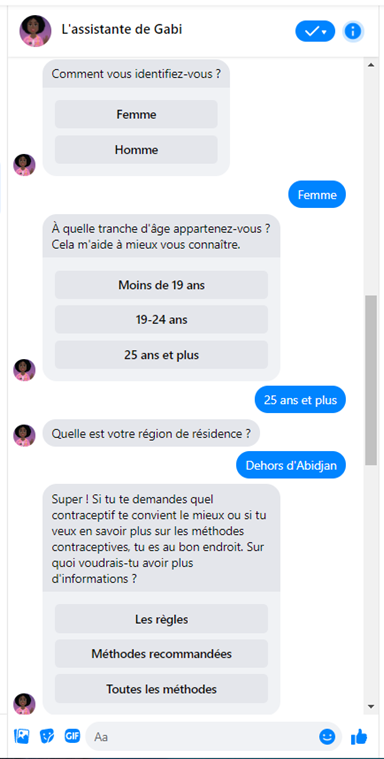
**

**Supplemental Materials 3:** Facebook advertisements for the Gabi chatbot


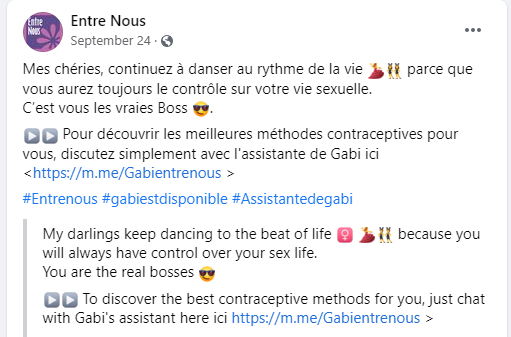

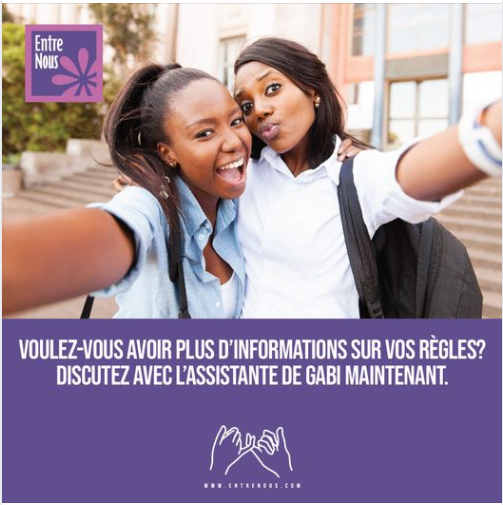

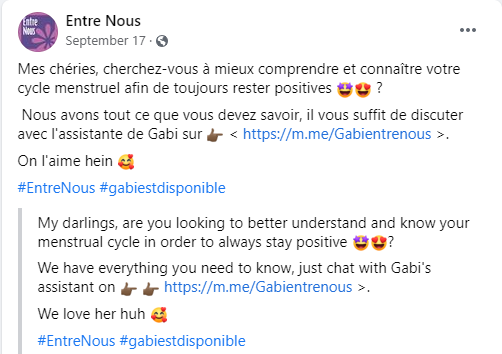

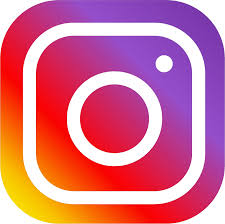

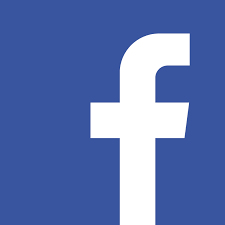

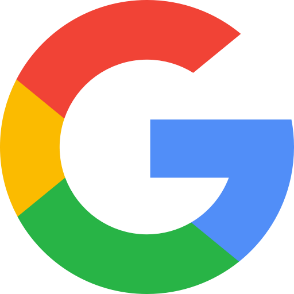


**Supplemental Materials 4:** Chatbot Backend User Journey (views/clicks)


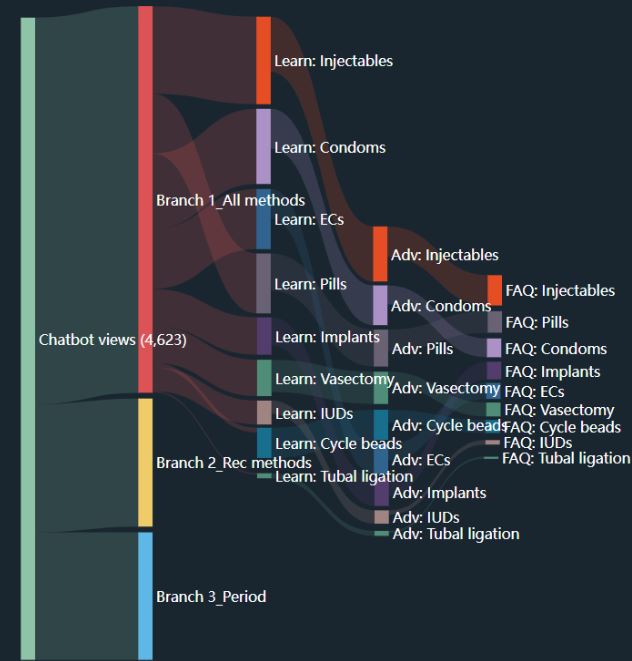

Supplement: Supplemental_Materials_1_to_4_oqae027 [file Supplemental_Materials_1_to_4_oqae027.docx]
